# Individualized Seizure Cluster Prediction Using Machine Learning and Chronic Ambulatory Intracranial EEG

**DOI:** 10.1109/TNB.2023.3275037

**Published:** 2023-10-03

**Authors:** Krishnakant V. Saboo, Yurui Cao, Vaclav Kremen, Vladimir Sladky, Nicholas M. Gregg, Paul M. Arnold, Philippa J. Karoly, Dean R. Freestone, Mark J. Cook, Gregory A. Worrell, Ravishankar K. Iyer

**Affiliations:** Department of Electrical and Computer Engineering, University of Illinois Urbana–Champaign, Urbana, IL 61801 USA; Department of Electrical and Computer Engineering, University of Illinois Urbana–Champaign, Urbana, IL 61801 USA; Mayo Clinic, Rochester, MN 55902 USA; Mayo Clinic, Rochester, MN 55902 USA; Mayo Clinic, Rochester, MN 55902 USA; Carle Foundation Hospital, Urbana, IL 61801 USA; Department of Biomedical Engineering, The University of Melbourne, Parkville, VIC 3010, Australia; Seer Medical Pty Ltd., Melbourne, VIC 3000, Australia; Department of Medicine, St Vincent’s Hospital Melbourne, University of Melbourne, Fitzroy, VIC 3065, Australia; Mayo Clinic, Rochester, MN 55902 USA; Department of Electrical and Computer Engineering, University of Illinois Urbana–Champaign, Urbana, IL 61801 USA

**Keywords:** Seizure clusters, relative entropy (REN), intracranial EEG (iEEG), seizure cluster prediction, bivariate feature

## Abstract

Epilepsy patients often experience acute repetitive seizures, known as seizure clusters, which can progress to prolonged seizures or status epilepticus if left untreated. Predicting the onset of seizure clusters is crucial to enable patients to receive preventative treatments. Additionally, studying the patterns of seizure clusters can help predict the seizure type (isolated or cluster) after observing a just occurred seizure. This paper presents machine learning models that use bivariate intracranial EEG (iEEG) features to predict seizure clustering. Specifically, we utilized relative entropy (REN) as a bivariate feature to capture potential differences in brain region interactions underlying isolated and cluster seizures. We analyzed a large ambulatory iEEG dataset collected from 15 patients and spanned up to 2 years of recordings for each patient, consisting of 3341 cluster seizures (from 427 clusters) and 369 isolated seizures. The dataset’s substantial number of seizures per patient enabled individualized analyses and predictions. We observed that REN was significantly different between isolated and cluster seizures in majority of the patients. Machine learning models based on REN: 1) predicted whether a seizure will occur soon after a given seizure with up to 69.5% Area under the ROC Curve (AUC), 2) predicted if a seizure is the first one in a cluster with up to 55.3% AUC, outperforming baseline techniques. Overall, our findings could be beneficial in addressing the clinical burden associated with seizure clusters, enabling patients to receive timely treatments and improving their quality of life.

## I. Introduction

Epilepsy is a neurological disorder that affects millions of people worldwide and is characterized by recurrent, unpredictable seizures that can significantly impact patients’ quality of life. Seizure clustering i.e., multiple seizures occurring within a short period, is a common phenomenon in people with epilepsy, with prevalence rates ranging from 13% to 76% [1]. Studies have shown that patients who experience seizure clusters have worse disease outcomes than those who do not [2]. Patients are often prescribed stronger anticonvulsant medications to manage the risk of seizure clusters, which may cause unwanted side effects [3]. These medications are given preemptively at the first sign of a seizure cluster (which may be the first seizure in many patients) since it is unknown whether another seizure will occur shortly after the termination of a seizure. Therefore, predicting whether a seizure will cluster, i.e., additional seizures occur shortly after the termination of the given seizure, is of great clinical importance.

Our goal is to develop individualized machine learning (ML) models for predicting seizure type, i.e., whether a seizure is an isolated seizure or part of a seizure cluster, using intracranial EEG (iEEG) data. iEEG data can be useful in creating models that offer precise and personalized predictions because it has the potential to capture differences in seizures of different types in patients with drug-resistant epilepsy [1], [3]. This paper extends our previous work [4], which demonstrated individualized models for predicting seizure type based on the differences in network interactions between isolated and cluster seizures. The current study further evaluates the clinical viability of cluster prediction by developing models for predicting cluster onset, studying the effects of training data size on prediction performance, and investigating the relationships between patient characteristics and model performance.

Predicting seizure types presents several challenges. Firstly, the difficulty of collecting long-term iEEG data with a sufficient number of isolated and clustered seizures for individuals presents a significant barrier in developing predictive models. Secondly, the differences in iEEG characteristics of isolated and cluster seizures are not yet fully understood. Third, there is limited exploration of machine learning (ML) methods that can be used for individualized cluster prediction. Although Ferastraoaru et al. identified differences in the duration of isolated and cluster seizures by pooling short-term data from 92 patients, they had limited data from each patient (3 − 31 seizures each) to explore patient-specific differences [5]. The patient-specific nature of seizure dynamics suggests that the differences between isolated and cluster seizures may be unique to each patient [6]. Karoly et al. found variations in the pre-ictal iEEG energy between isolated and cluster seizures in three out of 15 patients in the long-term NeuroVista data [7]. Chen et al. employed support vector machines to predict lead seizures (i.e., cluster onset) in canines using power in band features of inter-ictal long-term iEEG [8]. However, these studies did not investigate fine-grained iEEG features, such as bivariate features, which may further highlight patient-specific differences. To the best of our knowledge, previous studies have not demonstrated individualized iEEG-based seizure cluster prediction in humans.

Our prior research employed an innovative approach to address these gaps for predicting individualized seizure type using ML and a bivariate feature extracted from various physiologic frequency bands from long-term iEEG data [4]. (i) We studied various frequency bands to capture patient-specific bands with prominent seizure-related iEEG changes [9]. (ii) Since epilepsy is a network disorder [10], [11], we employed a bivariate feature, relative entropy (REN), to quantify the network interactions underlying different seizure types. (iii) We used individualized long-term iEEG data to capture a sufficient number of seizures in each patient to explore patient-specific differences in dynamics [6] and train individualized models. Finally, (iv) we trained various linear and non-linear ML models using REN data as features to predict seizure type.

Here, we extended our approach to predict the onset of seizure clusters, which is important for administering appropriate treatment to prevent progression [12]. Furthermore, we investigated the minimum training sample size required to achieve optimal performance of the individualized models. This could be beneficial in pre-surgical iEEG monitoring when a limited number of seizures are often recorded [13]. Patients with implanted iEEG recording devices can also benefit from earlier predictions that require less training data. Lastly, we explored the association between patient characteristics (demographics, disease duration, etc.) and model performance to guide the selection of patients who could benefit from the development of individualized prediction models.

We evaluated our approach using NeuroVista data [14], one of the largest chronic ambulatory iEEG datasets, consisting of data from 15 patients with up to 2 years of recordings for each patient. The analysis included 3710 isolated and cluster seizures. Seizures within 24 hours of each other were considered to belong to a cluster. For each seizure, iEEG data from the ictal and 10 minutes pre-ictal period (*near-seizure*) were used. We computed REN from the iEEG data since it has previously been used for seizure detection [15] and seizure onset zone localization [16]. To evaluate whether REN captures differences in seizure types, averaged REN values in different frequency bands for isolated and cluster seizures were statistically compared [6]. Based on insights from the statistical analysis, we developed several individualized ML-based prediction models (i) to predict whether the subsequent seizure will occur shortly in the future after the given seizure, and (ii) to predict cluster onset. We compared ML models’ performance with baseline techniques. Finally, we assessed the robustness of our results by repeating the analyses with seizures within 8 hours of each other being considered as part of a cluster.

**Fig. 1.**
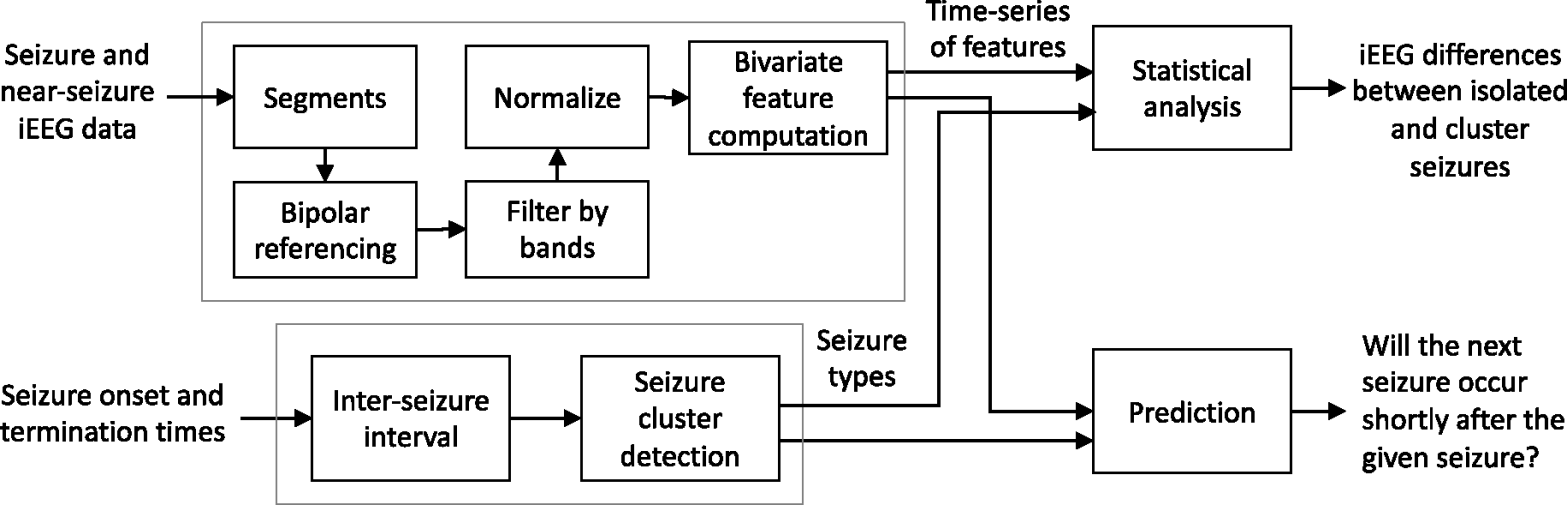
Analysis pipeline. iEEG data was pre-processed to extract bivariate iEEG features. The ground truth information of seizure types along with the features was used for statistical analyses and machine learning modelling.

Our contributions are as follows:

1. We proposed a generic framework (Figure 1, Figure 3) for investigating the dynamics of cluster seizures and for predicting if a given patient will experience a seizure cluster or an isolated seizure.
2. Using REN from various frequency bands, we found significant differences between the dynamics of isolated and cluster seizures in six patients, substantially improving over a previous approach that found differences only in three patients on the same dataset.
3. Majority of the differences were in the beta and gamma bands and during the near-seizure period. Compared to isolated seizure, near-seizure REN was higher and ictal REN was lower for cluster seizures.
4. Individualized ML models predicted subsequent seizure onset in the near future with 69.5% AUC and predicted cluster onset with 55.3% AUC, outperforming baseline predictors. Patient characteristics were not associated with the prediction performance.
5. We observed that the minimum number of training sampled needed to achieve optimal ML performance for seizure type prediction varied across patients.
6. The results were robust to change in the inter-seizure interval threshold for clustering.

The models can be clinically valuable in guiding the selection of anticonvulsant medications based on seizure type. Moreover, insights into the dynamics of seizure clusters can guide the exploration of techniques to mitigate their clinical burden, e.g., through changes in brain stimulation parameters.

## II. Methods

The overall analyses pipeline is shown in Figure 1. The details of each step are described below.

### A. Long-Term iEEG Data Collection

We used data that was collected as part of the NeuroVista study in Australia [14] (Clinical Trial number - NCT01043406). In that study, 15 patients with refractory epilepsy were implanted with an intracranial EEG device that collected data in an ambulatory setting for up to 2 years in each patient. Data from six patients was excluded from this study due to significant data drops which could affect the analysis of clustering. The nine patients included in the study had an average recording duration of 550 ± 208 days. Data for each patient consisted of iEEG data collected from 16 electrodes (2 lead assemblies with 8 contacts distributed across 2 electrode arrays each) placed on the presurgically assessed seizure onset zone. Data was sampled at 400Hz and wirelessly transmitted from the implanted device to an external, hand-held personal device.

### B. Seizure Detection and the Selection of Near-Seizure Data

Seizures were detected from the iEEG data for each patient using a published methodology [17]. This procedure resulted in an average of 412.2 ± 348.4 seizures per patient over the course of their entire recording. The average duration of the seizures was 39.0 ± 63.6s. Since pre-ictal activity near seizure onset can show differences between different seizure types [7], we included pre-ictal iEEG in our analysis. For each seizure, pre-ictal iEEG up to 10 minutes prior to seizure onset was considered because seizure-related changes can manifest in that duration [18]. In case a seizure had occurred within the previous 10 minutes of the given seizure, the iEEG data between the termination of the previous seizure and the onset of the given seizure was considered. We refer to this pre-ictal iEEG data as *near-seizure*.

### C. iEEG Preprocessing

iEEG data was preprocessed as follows. First, since ambulatory recordings can have several artifacts, we used a bipolar montage for referencing the signals. Bipolar montage was computed by taking the difference between the iEEG signals on consecutive channels on each array, resulting in 12 bipolar pairs (2 lead assemblies × 2 arrays × 3 bipolar pairs per array) per patient. For clarity, we refer to each bipolar pair as an “electrode” for the remainder of the analysis unless otherwise stated. Seizure and near-seizure iEEG data from each electrode was divided into 2.5s non-overlapping segments. Segment length was chosen as 2.5s to provide a sufficient number of samples in each segment to robustly estimate relative entropy. Segments in seizure and near-seizure periods were aligned to seizure onset. Additional artifact removal was not done because the iEEG data used for the analysis was of a short duration and since bipolar montage can remove artifacts that are common across channels. Data in each segment was filtered into the following physiologic bands for feature extraction: delta (0.5 – 4Hz), theta (4 – 8Hz), alpha (8 – 12Hz), beta (12 – 25Hz), and gamma (25 – 45Hz) using 2^nd^ order Butterworth bandpass filters. The resulting timeseries for each band and electrode within a segment was independently normalized to have zero mean and unit variance.

### D. Seizure Cluster Detection

Seizure clusters were identified based on the inter-seizure intervals (ISIs) of successive seizures. Several definitions for seizure clusters have been proposed in literature based on a cutoff for the ISI, ranging from 2 hours to 24 hours [5], [19]. For this analysis, we used 24 hours as the cutoff since it is widely used. Based on this definition, seizures were categorized into the following three types: (i) *Isolated* seizures were seizures that did not have a seizure 24 hours before or after them. The remaining seizures were categorized as cluster seizures. Cluster seizures were further categorized into (ii) *cluster-last*, which were the last seizures in clusters, and (iii) *cluster-non last*, which were seizures in a cluster that were not the last seizure. Our analyses also considered (iv) *cluster-first* seizures, which were the first seizures in clusters. Seizures within clusters were subcategorized to study differences and similarities between those subcategories and isolated seizures. Near-seizure iEEG segments were considered to be in the same category as their corresponding seizure. To assess the robustness of our results to the definition of seizure clusters, we also evaluated all the results for an 8 hour ISI cutoff.

### E. Bivariate Feature Computation

**Fig. 2.**
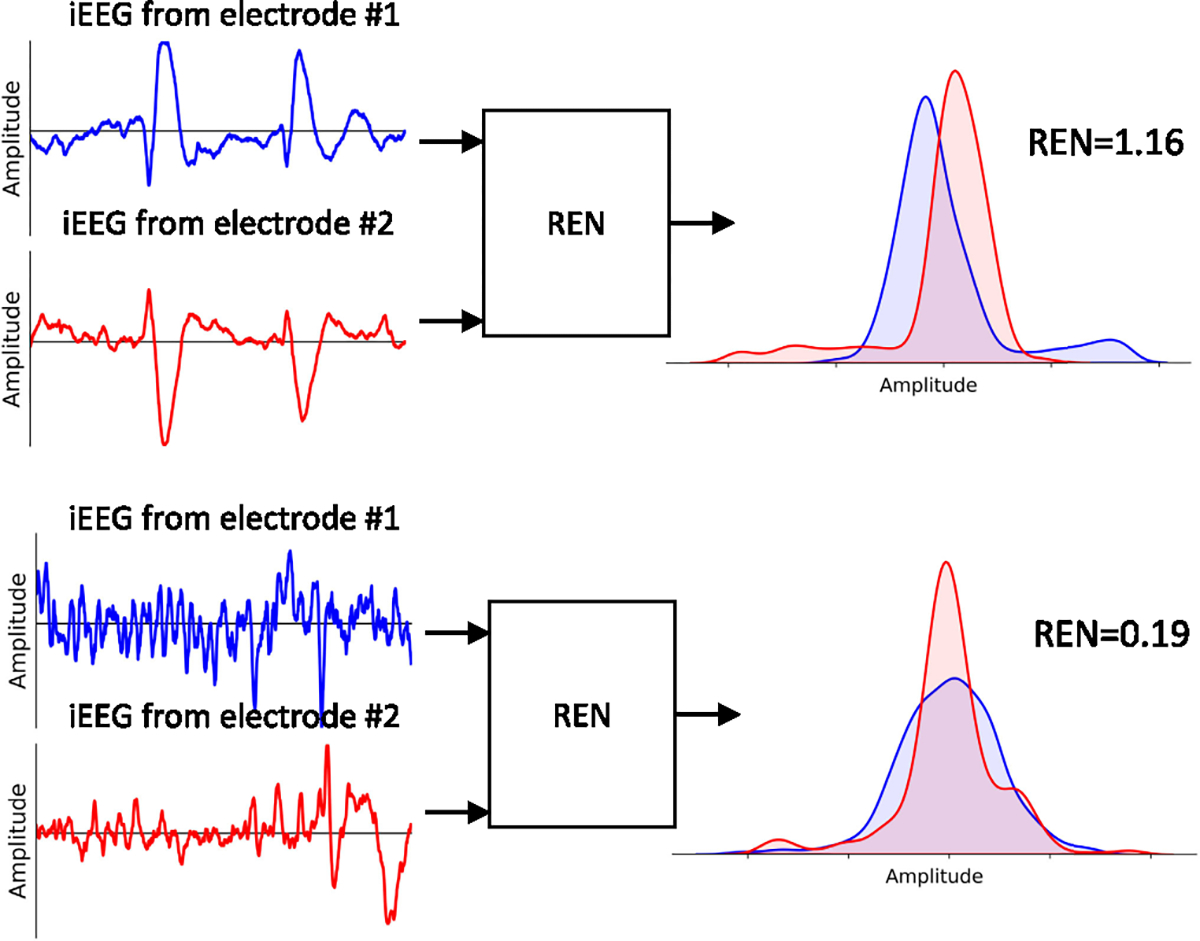
Examples of REN computation. Panels show the normalized signals, the distributions of their amplitudes, and the resulting REN value. The upper panel shows a pair of signals that have a higher REN value than the signals in the lower panel.

REN is a bivariate feature and quantifies the dissimilarity in the distribution of iEEG signal amplitudes between two electrodes. REN was computed for each segment and band separately. For a pair of electrodes, the distributions of amplitudes of the filtered signals were compared using KL divergence (Figure 2). Since KL divergence is nonsymmetric, the dissimilarity was measured with each signal as reference and the maximum was considered as REN [16]. REN was computed for all pairs of electrodes within a patient.

### F. Statistical Analyses

We compared REN of different types of seizures separately for each band. Since we were primarily interested in differences between isolated and cluster seizures, in each scenario three comparisons were done: (i) isolated vs cluster-first, (ii) isolated vs cluster-non last, and (iii) isolated vs cluster-last. Wilcoxon rank sum test was used for statistical comparison, and FDR correction was applied to correct for multiple testing.

### G. Machine Learning Prediction Models

**Fig. 3.**
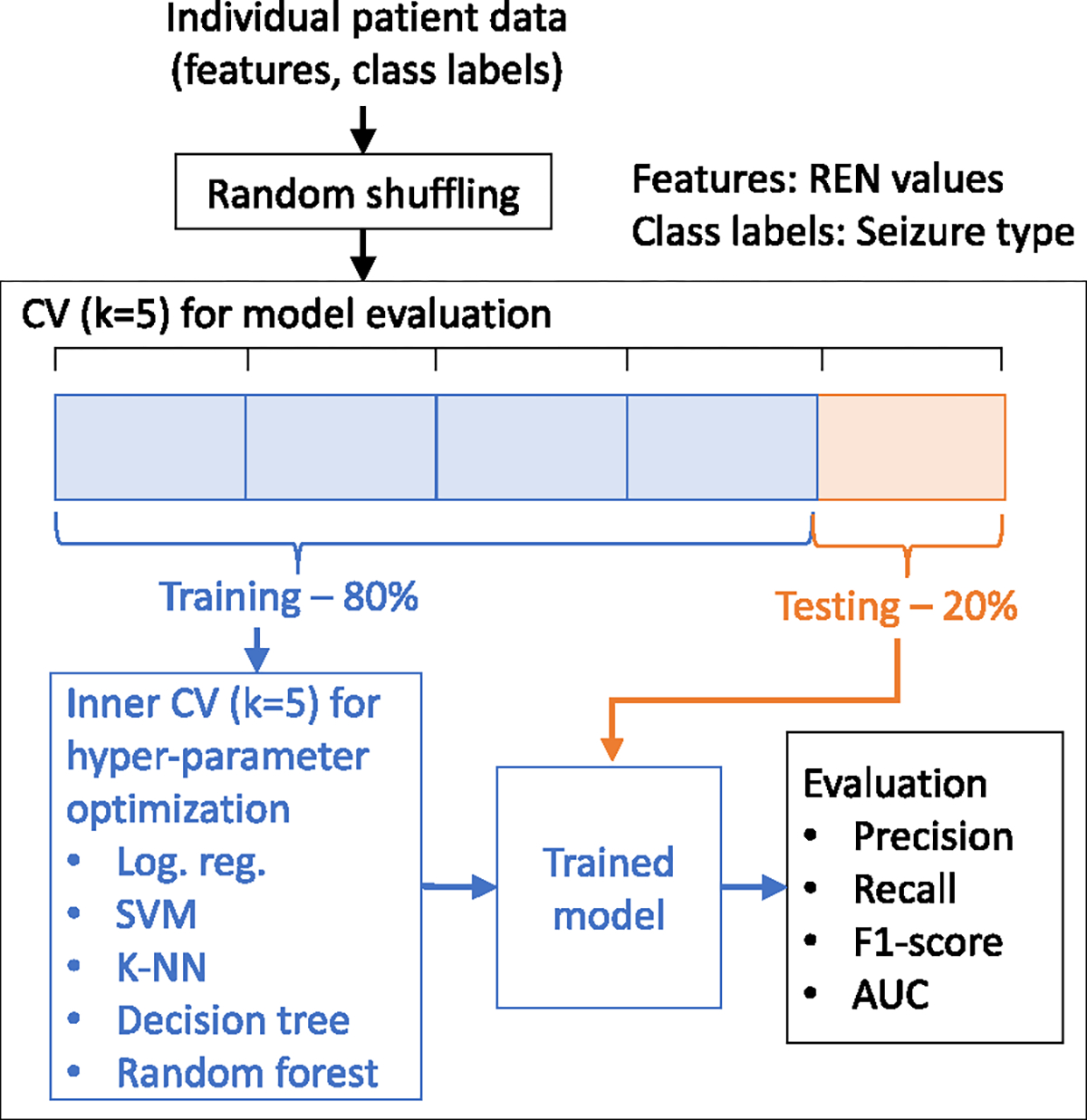
Workflow for developing and evaluating seizure cluster prediction models. Abbr: CV, cross-validation; log. reg., logistic regression.

We tested several linear and non-linear classification methods for predicting seizure clustering (Figure 3). The linear models evaluated in this study were logistic regression and support vector machine (SVM). Among non-linear classifiers, random forest, decision trees, and k-nearest neighbors (k-NN) were evaluated. For each classifier, we used 5-fold stratified cross validation with 80%–20% training-testing split of the patient’s data. Classifiers were individualized by only training and testing on data of the same patient. Since each method had several hyper-parameters that could affect model performance, we used inner 5-fold stratified cross validation for selecting hyper-parameters using only the training data (which was split into training and validation sets). Hyper-parameters for each classifier were the same as in the previous study [4]. Due to imbalance in class sizes, samples were weighted inversely proportional to class size during model training. We used precision, recall, F1-score, and area under the receiver operating characteristics curve (AUC) as performance metrics.

We considered two prediction tasks. (1) *Next seizure prediction*: For the first task, we used average REN values from the different bands and periods as input and predicted whether the next seizure would occur within 24 hours of the given seizure or not. For this task, seizures were divided as follows: (i) “isolated + cluster-last” - isolated seizures and the last seizure in clusters, since no seizure occurs shortly after them; and (ii) “cluster-non last” - the remaining seizures in clusters. (2) *Cluster onset prediction*: For this task, we used average REN values from the different bands and periods as input and predicted whether the given seizure was an isolated seizure or the first seizure in a cluster. For this task, seizures were divided as follows: (i) “isolated” - isolated seizures since no seizure occurs shortly after them; and (ii) “cluster-first” - the first seizure in clusters because it indicates the onset of a cluster. During robustness analysis, the same classes were used although the model predicted whether the next seizure would occur within 8 hours of the given seizure or not.

We compared the models with two baseline predictors: (i) a chance-level predictor (*baseline 1*), and (ii) a model that always predicts “cluster”, i.e., another seizure will occur soon (*baseline 2*). The theoretical performance for the baseline predictors were calculated as follows. Assume that the true probability of a seizure to be cluster-non last is r, and the model predicts the cluster-non last label with probability q. Then, the precision is r for both *baseline 1* and *baseline 2*. The recall is q for *baseline 1* and 1 for *baseline 2*. Thus, the F1 score for *baseline 1* is $\frac{2rq}{r+q}$, and the F1 score for *baseline 2* is $\frac{2r}{r+1}$. The AUC for *baseline 1* is 0.5 and the AUC for *baseline 2* can not be computed. The same approach was used to derive baseline performance for cluster-onset prediction, with r and q representing the true probability and prediction probability of a seizure being cluster-first, respectively.

### H. Effect of Sample Size on Prediction Performance

To understand the effect of sample size on the prediction performance, we artificially reduced the training set size and trained ML models with the reduced dataset. We divided the entire data into a fixed test set (20%) and a training set (80%). We sampled x% of the training set to train the model, with x∈{20,40,60,80,100}. Sampling was repeated five times for each x to account for sampling bias. Model performance was evaluated on the same complete test set for different sizes of the training data. Since the number of seizures varied substantially across patient, the percentage of training set used for model training doesn’t allow for a holistic comparison across patients. Therefore, in additional experiments, we sampled $2^{y}$ samples from the training set to train the model, with y∈{4,5,6,7,8,9}, and tested them the same way as given above. These analyses were restricted to the next cluster prediction task with a 24 hr ISI threshold.

## III. Results

### A. Patient Characteristics and Seizure Type Distribution

**Fig. 4.**
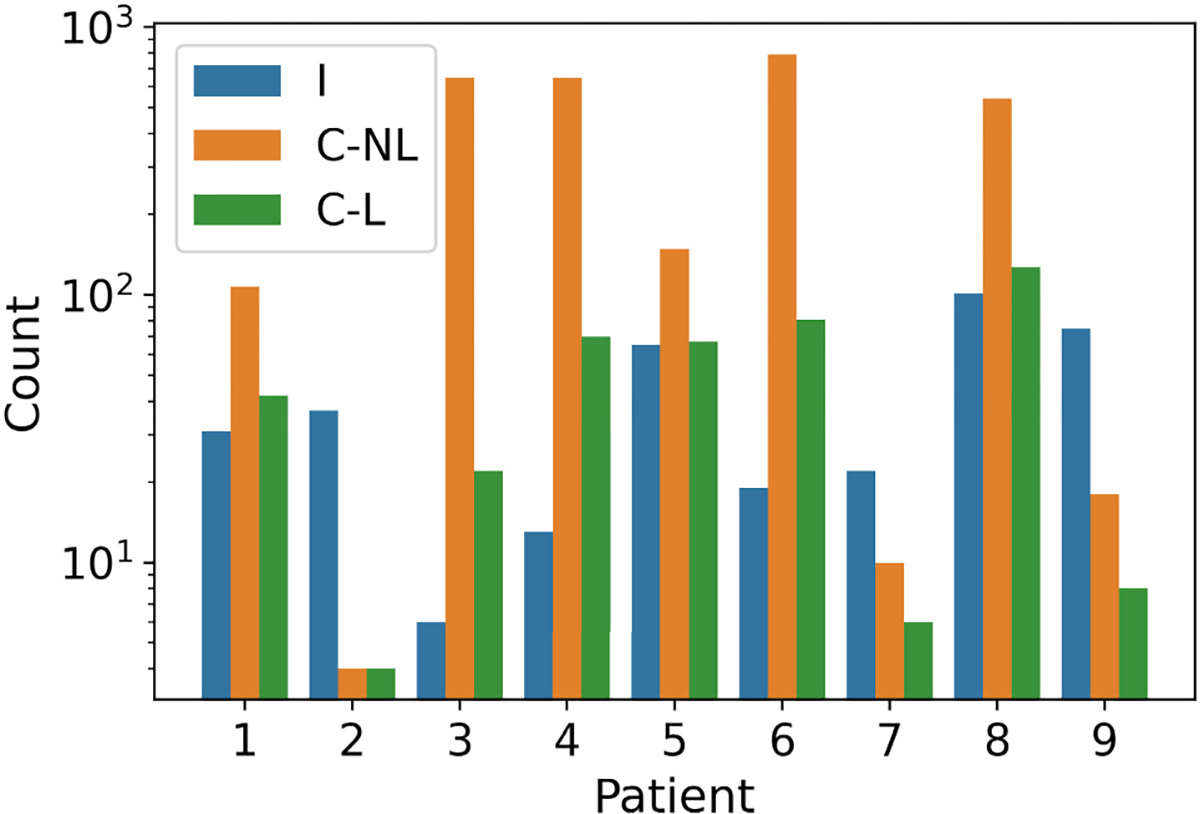
Data summary. Number of seizures of each type in each patient for an ISI threshold of 24 hours. Note that cluster-first seizures are not shown in the plot because the number of cluster-first seizures is the same as the number of cluster-last seizures. Abbreviations: I, isolated seizure; C-L, cluster-last seizure; C-NL, cluster-non last seizure.

Majority of the patients were male, were diagnosed with epilepsy by the age of 20 years, and had not undergone resection surgery previously (Table I). The epileptogenic zone was in different parts of the brain across patients. We observed seizures of both types in all the patients (Figure 4). The ratio of isolated to cluster seizures varied from 0.01 – 4.63 across patients. For example, patients #3, #4, and #6 had very few isolated seizures compared to cluster seizures, whereas, patient #9 had many more isolated seizures than cluster seizures. Across all patients, there were a total of 369 isolated seizures, 2914 cluster-non last seizures, and 427 cluster-last seizures.

### B. Patient-Specific Differences in Grand Average REN

**Fig. 5.**
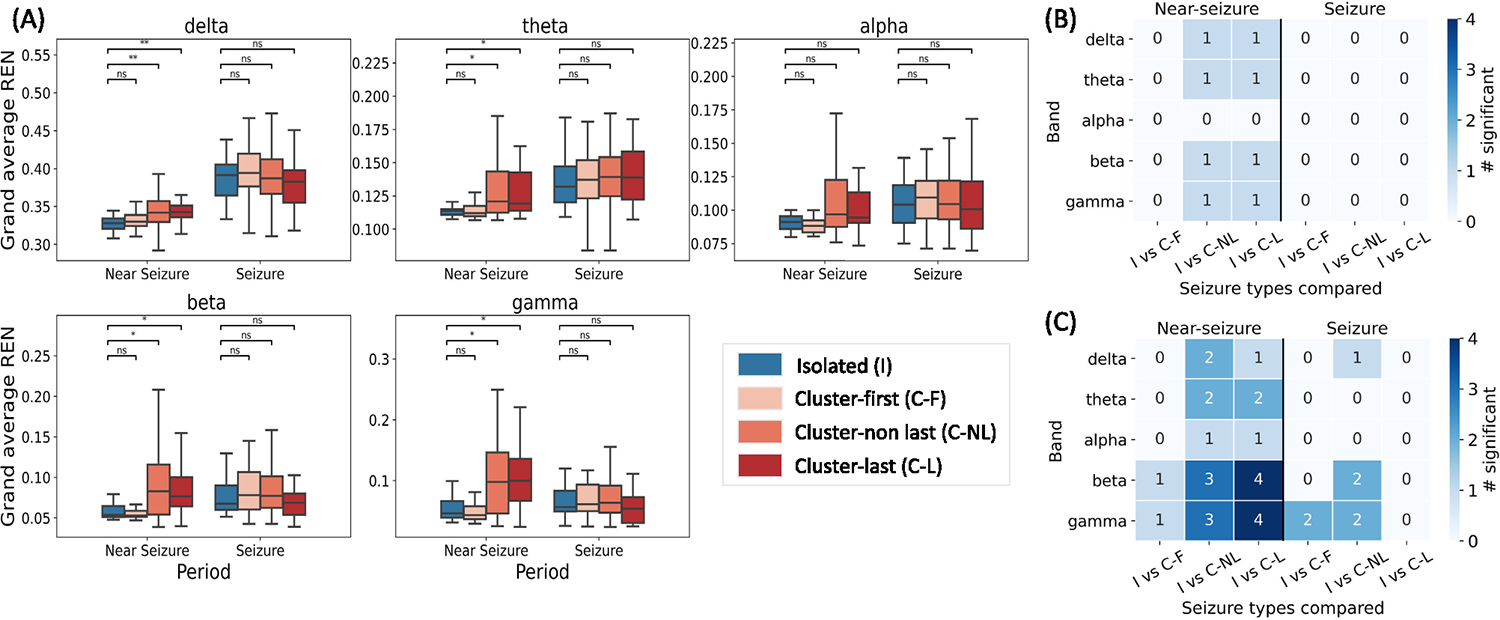
Comparison of grand average REN values with a 24 hours cutoff for ISI. (A) Grand average REN values for different seizure types, bands, and periods for patient #1. Statistical comparison between seizure types during near-seizure and seizure periods is shown (* denotes p<0.05, ** denotes p<0.001). FDR correction was applied for all comparisons across patients. (B) Significant differences between grand average REN values in patient #1 represented as a heatmap. (C) Significant differences in grand average REN aggregated across patients. Annotations show the number of patients in whom there were significant differences. Abbr: I, isolated seizure; C-NL, cluster-non last seizure; C-L, cluster-last seizure.

To understand whether there were differences in the dynamics of different seizure types, we compared the grand average REN values within each patient. To obtain the grand average REN, REN values for all pairs of electrodes and all segments within a band and period were averaged separately for each seizure. In patient #1, there were differences in the delta, theta, beta, and gamma bands in the near-seizure period, but not during seizure, for comparisons between isolated vs cluster-last and isolated vs cluster non-last seizures (Figures 5A, 5B). No significant differences were observed between isolated and cluster-first seizures for patient #1.

Aggregating the grand average REN comparisons from all patients showed that the majority of the differences were seen near-seizure, especially in the beta and gamma bands (Figure 5C). There were no significant differences between isolated and cluster-last seizures during the ictal period. Differences between isolated and cluster-first seizures were observed only in four instances across two patients.

Interestingly, patient-specific significant differences in grand average REN were observed in six out of nine patients in at least one band, duration, and seizure type comparison. No significant differences were observed in patients #2, #3, and #4, all of whom had very few seizures of at least one type. Patient #2 had very few cluster seizures (4 non-last, 4 last), whereas patients #3 and #4 had very few isolated seizures (<20) compared to cluster seizures (>600; Figure 4).

**Fig. 6.**
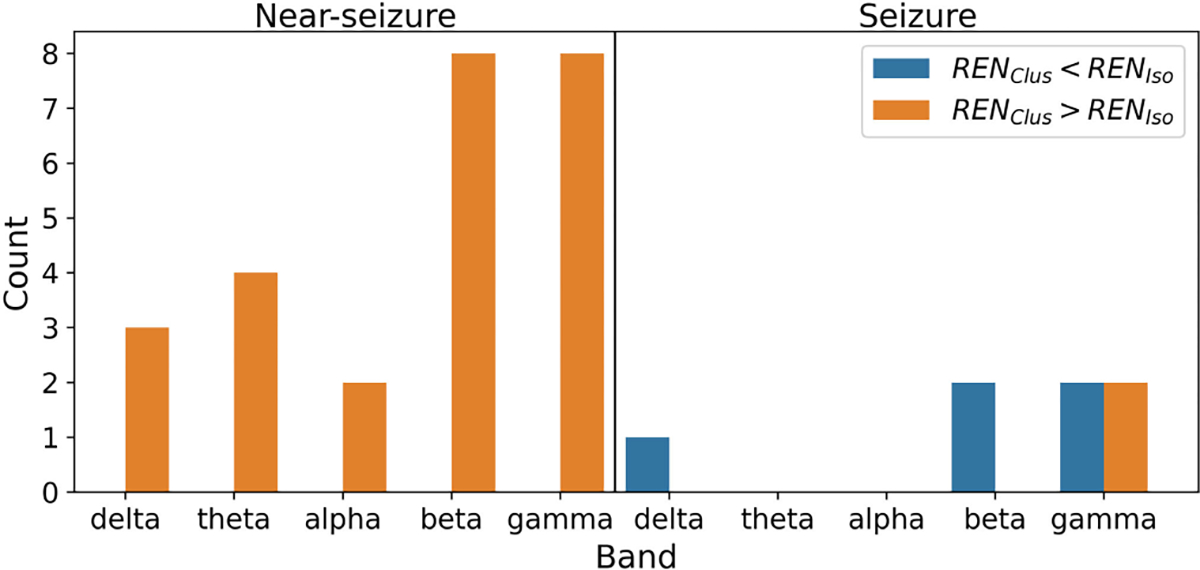
Number of cases in which REN was higher for isolated seizures (in blue) or higher for clustered seizures (in orange) with a 24 hour cutoff for ISI. Only cases with a significant difference between seizure types were considered (Figure 5C). Count was obtained by aggregating across patients and seizure type comparisons.

We also studied which seizures had higher grand REN (Figure 6). This analysis was restricted to the cases in which REN was significantly different between isolated and clustered seizures. During the near-seizure period, grand average REN was higher for the clustered seizures than the isolated seizures. On the other hand, during seizures, grand average REN values were typically lower for cluster seizures than isolated seizures.

### C. Next Seizure Prediction

Grand average REN from different bands and periods were used for prediction because they were significantly different between seizure types in a majority of patients. This resulted in 10 features for each seizure (5 bands × 2 periods). Patient #2 was excluded from the prediction analysis because they had very few cluster-non last seizures (n=4, Figure 4). On average across the remaining eight patients, random forests achieved the best prediction (Table II) with 69.5% AUC while k-NN achieved the best precision of 80.9 % and best F1-score of 78.8%. Performance of a majority of the prediction models was comparable or better than the baseline models.

We provide the patient-specific performance for random forest because it achieved the best AUC (Table III). The performance varied across patients, ranging from 54.2% − 94.9% F1-score. Random forest was better than chance level predictor (baseline 1) for all the patients based on F1-score. Random forest had higher F1-score than baseline 2 (that always predicts “cluster”) in two patients. Although baseline 2 achieved a higher F1-score for patients with a higher number of cluster seizures, it produced a considerable number of false alarms. ML models reduced the number of false alarms, as demonstrated by their higher precision (Table III).

We evaluated whether the prediction performance was dependent on the patient characteristics. Patients were grouped based on variables in Table I and AUC values of patients from different groups were statistically compared using a Wilcoxon rank-sums test. For continuous variables, we tested for correlation with the variable and the AUC. None of the patient characteristics – sex, epileptogenic zone, previous resection, age, age of diagnosis, or disease duration – were associated with the prediction performance.

**Fig. 7.**
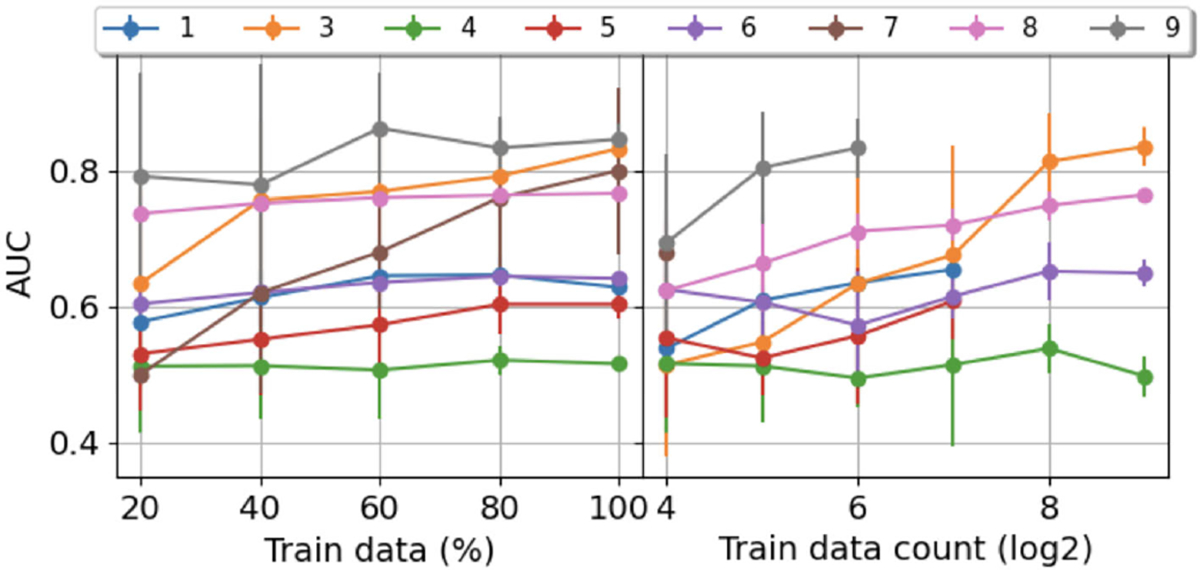
AUC on the test set of models trained with different number of samples for the next seizure prediction task with a 24 hour ISI cutoff. Sampling was performed five times for each scenario, with the means plotted in dots, and standard deviations plotted as vertical lines. Each color represents an individual patient.

We also explored the impact of sample size on prediction performance by varying the number of training samples (Figure 7). Increasing the training set size boosted model performance. However, we only observed a substantial improvement in the prediction performance of patient #7. We speculate that this was because the other patients had considerably more seizures than patient #7, hence, 20% of their data enough to train the model. When we examined the performance changes while increasing the number of training samples exponentially, we observed that model performance increased considerably across patients. The minimum number of samples required to achieve an optimal AUC ranged from 64 to 256 across patients.

### D. Cluster Onset Prediction

We used the 10 grand average REN from different bands and periods for predicting cluster onset. Patients #2, #3, #7, #9 were excluded because they had very few isolated or cluster-first seizures (n<10, Figure 4). SVM achieved the best prediction (TableIV) with 70.4 % precision and 55.3 % AUC. Performance of a majority of the ML models was comparable or better than the baseline models.

The performance for SVM varied across patients, ranging from 60.6% − 78.5% F1-score (Table V). SVM was better than chance level predictor (baseline 1) for all the patients based on F1-score. Similar to the next seizure prediction task, although baseline 2 had better or comparable F1-score as SVM, it produced more false positives than SVM.

The performance for next seizure prediction (Table II) was better than cluster onset prediction (Table IV) based on the AUC. This concurs with the statistical analysis (Figure 5) in which more differences were observed between isolated vs cluster non-last seizures than between isolated vs cluster-first seizures. Non-linear ML models were the best performing models for the next seizure prediction task whereas linear models achieved better performance for cluster onset prediction. It is possible that linear models fit the data better because fewer samples were available for cluster onset prediction.

### E. Robustness Analyses

**Fig. 8.**
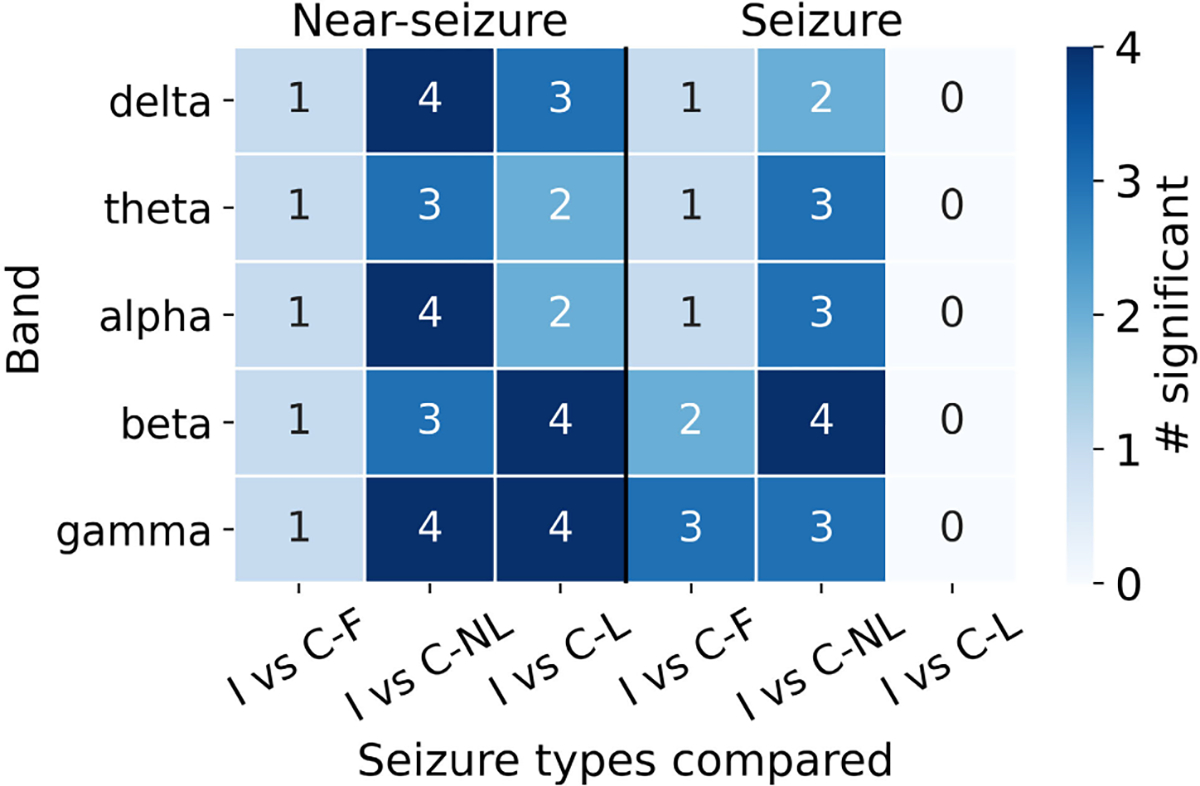
Significant differences in grand average REN aggregated across patients with an 8 hours cutoff for ISI. FDR correction was applied for all comparisons across patients. Annotations show the number of patients in whom there were significant differences. Abbreviations: I, isolated seizure; C-NL, cluster-non last seizure; C-L, cluster-last seizure.

**Fig. 9.**
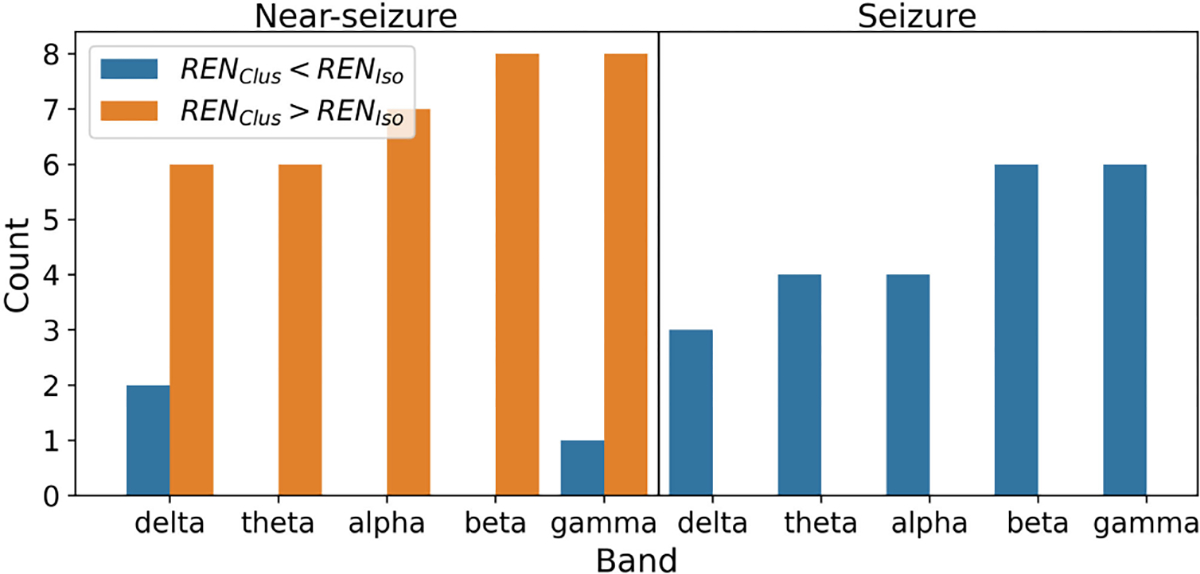
Number of cases when REN was higher for isolated seizures (in blue) or higher for clustered seizures (in orange) with an 8 hour cutoff for ISI. Only cases with a significant difference between seizure types were considered (Figure 5C). Count was obtained by aggregating across patients and seizure type comparisons.

We evaluated the robustness of our results to the definition of clusters by repeating the analyses using an 8 hour ISI cutoff. Overall, the results with the modified cutoff were largely consistent with the 24 hours cutoff results. There were 948 isolated clusters, 2123 cluster-non last seizures, and 639 cluster-last seizures. Grand average REN was significantly different in eight patients (Figure 8). Majority of the differences were observed in the beta and gamma bands. There were no differences in grand average REN between isolated and cluster-last seizures during the ictal period. Isolated and cluster-first seizures were significantly different for four patients (#4, #6, #7, #8). Fewer differences were observed between isolated vs cluster-first seizures than isolated vs cluster-non last seizures. During the near-seizure period, REN was typically higher for the clustered seizures than the isolated seizures but the reverse was observed during seizures (Figure 9).

Patients with less than 10 seizures of any category were removed for the prediction analyses – patients #2 and #7 for next seizure prediction and patients #2, #7, and #9 for cluster onset prediction. For the next seizure prediction task, random forest models were the best predictors with 70.3% AUC averaged across patients (Table VI). Random forest also achieved the best AUC of 64.2 % in predicting cluster onset (Table VII). ML techniques were better than or comparable to baseline predictors for both prediction tasks.

## IV. Related Work

We discuss related work from: (i) seizure forecasting, which tackles a similar problem of predicting the next seizure and has motivated the use of different features in our analysis; (ii) seizure cluster detection and analyses, which have highlighted salient characteristics of cluster seizures, and (iii) seizure cluster prediction, which have used long term iEEG and non-iEEG data from canines and humans to predict clustering.

### A. Seizure Forecasting

Seizure forecasting considers the problem of predicting the likelihood of a seizure at a given time in the future based on inter-ictal and pre-ictal data. Several techniques have been developed for seizure forecasting, ranging from traditional ML methods to more recent deep learning models [20], [21] Univariate, bivariate, and multivariate features extracted from inter-ictal EEG data have been used for seizure forecasting with varying degrees of success [22], [23]. CNNs applied to EEG forecasted seizures in canines and humans better than hand-crafted features combined with traditional ML methods [21], [24]. Our approach differs from the forecasting literature in the use of pre-ictal and ictal data to predict seizures, while a majority of forecasting models use inter-ictal data only.

### B. Seizure Cluster Detection and Comparison Analyses

Previous methods have mainly addressed the detection of seizure clusters retrospectively based on inter-seizure intervals (ISI) using threshold-based methods and statistical methods [25]. Threshold based methods classify seizures with ISI less than the given threshold (for e.g., 8 hrs, 24hrs) as belonging to a cluster [5]. While these methods are easy to use, they do not account for the differences in baseline seizure rate of individuals and can be prone to false positives/negatives. On the other hand, statistical methods rely on trends in the data to identify clusters. Chiang et al. proposed a change point detection-based method that relies on seizure diaries to identify seizure clusters and identified several clusters that were missed by threshold detectors [19]. For the NeuroVista data, Seneviratne et al. visualized trends in ISI to identify seizure clusters and seizure bursts [26]. Most cluster detection methods rely on ISI for detection and are, therefore, not suitable for the proposed prediction task.

Few studies have statistically compared isolated seizures and seizure clusters to identify differences in their characteristics. Ferastraoaru et al. compared the duration of isolated and cluster seizures pooled from 92 patients and observed that isolated seizures were longer than the first seizure in a cluster and intracluster seizures, but were similar in duration to the last seizure in a cluster [5]. Karoly et al. compared isolated seizures and cluster seizures with very short ISI, termed as seizure bursts, in the NeuroVista data [7], and observed differences in the energy in the pre-ictal period of isolated seizures and burst seizures in some patients. Previous studies have not compared seizure type using a bivariate iEEG feature.

### C. Seizure Cluster Forecasting and Prediction

Ilyas et al. used long-term seizure timing data to forecast seizure clusters in humans [27]. They built individualized autoregressive models that predicted the probability of seizure clustering, derived from the Kolmogorov-Smirnov statistic, and demonstrated better than chance performance in cluster forecasting. Another recent study predicted lead seizures (i.e., cluster onset) using long-term iEEG data in canines [8]. They used univariate power in band features from pre-ictal and interictal iEEG combined with SVM to predict the onset of clusters and achieved high accuracy. Our approach of using ictal iEEG data to predict the next seizure provides a complementary technique to existing approaches.

## V. Discussion

We leveraged a bivariate iEEG measure applied to long-term iEEG to discover differences in the seizure dynamics of isolated and clustered seizures and to predict individualized seizure clustering. The dynamics for isolated and cluster seizures were different in six out of nine patients. The majority of the differences were observed in the higher frequency bands (beta and gamma) and in the pre-ictal (near-seizure) period. Patient-specific ML models based on REN achieved 69.5% AUC in predicting clustering, i.e., the occurrence of another seizure shortly after a seizure, and were only slightly better than chance (55.3% AUC) in predicting cluster onset. Our approach can be clinically valuable in personalizing epilepsy treatment by guiding the selection of anticonvulsant drug suitable for a given seizure type. Our approach also supports the application of graph-theoretic methods [9], [28] to gain further insights into seizure progression of different seizure types, which can be useful in predicting seizure clusters.

### Fine-Grained iEEG Features are Valuable for Differentiating Seizure Type Dynamics:

Karoly et al. [7] found differences in the energy of pre-ictal iEEG in three out of 15 patients in the same dataset. In contrast, we observed differences in six out of nine patients by analysing different physiological bands separately and using REN, which captures network-level interactions between electrodes. These could have provided a greater resolution for differentiating seizure type dynamics. The majority of the differences were observed in the beta and gamma bands, whose contribution to iEEG energy is substantially lower than delta and theta bands that showed similar dynamics for the different seizure types. REN was higher for cluster seizures than isolated seizures in the near-seizure period but lower during the seizure period, suggesting that the network interactions underlying cluster and isolated seizures are different. Further investigation of these bands could provide insights into the network interactions that increase the propensity for cluster seizures.

### Long-Term Data on Individuals is Vital to Characterize and Predict Seizure Type:

It is critical to use iEEG data of a large number of seizures from a patient to study the dynamics of different seizure types due to (i) patient-specific differences in the dynamics and (ii) the variation in iEEG characteristics of seizures across patients. Sensitivity to seizure numbers is also supported by our statistical and machine learning analyses. The three patients for whom we observed no differences across seizure types had few seizures (< 20) of at least one type. Long-term data with sufficient number of seizures is also crucial for developing individualized prediction models. Our results demonstrated an increase in model performance when training with more samples. Since the minimum number of samples required to achieve optimal performance ranged from 64 to 256 across patients, presurgical iEEG recordings may not provide sufficient samples for optimal model training [13].

### Pre-Ictal Dynamics of Cluster-Last Seizures are Different From Isolated Seizures:

Inspired by Ferastraoaru et al. [5], we subcategorized cluster seizures into cluster-last and cluster-non last seizures for comparison with isolated seizures. They found that isolated seizures were similar in duration to cluster-last seizures [5]. In line with their result, we observed no differences in grand average REN during seizure period between isolated and cluster-last seizures. However, we also observed significant differences between isolated and cluster-last seizures during the near-seizure period. Our results suggest that onset mechanisms of isolated and cluster-last seizures may be different. Future work studying differences in the dynamics of cluster-last and cluster-non last seizures can be useful in predicting the end of a seizure cluster.

### Challenges in Predicting Seizure Clustering:

Although the current results uniquely demonstrate the possibility of predicting seizure clusters, improvement in the performance is needed for clinical utility. Limited number of samples (isolated and cluster seizures) and data imbalance present major challenges in the application of sophisticated ML methods. There is a considerable overlap between the REN values of cluster and isolated seizures, which further affects prediction performance and motivates the need for better iEEG features. The similarity in REN between isolated and cluster-first seizures made it difficult to predict cluster onset, as has been observed in previous studies [29]. Future studies may consider these challenges for improving prediction.

### Limitations and Future Work:

There are several limitations of our study related to design choices, data aggregation, and ML analyses. Firstly, we used a threshold-based approach for detecting seizure clusters. It has been argued that differences in the baseline rate of seizures of individuals must be taken into account for detecting seizure clusters [19]. Secondly, seizure onset zone and non-seizure onset zone electrodes have different dynamics [16] although we did not distinguish between them in the current study.

Thirdly, we averaged REN values across time segments, thus smoothing out temporal trends in REN which could be indicative of seizure progression and type [30]. A challenge in comparing temporal trends in REN across seizures is the variability in seizure duration. Methods to transform timeseries of varying lengths to the same length have been previously used for analysing seizures and can be useful [28], [31].

Finally, further improvements are required in the prediction performance. Advanced ML methods that can learn complex short- and long-term relationships in REN timeseries and that pool data across patients to improve sample size may boost predictive performance [32]. We plan to address these limitations in future work.

## Figures and Tables

**TABLE I:** Patient Characteristics. Note That Patient Numbering Is Different From the Original Trial [14]

Pat. #	Age (yrs)	Diagnosis Age (yrs)	Sex	Epileptogenic Zone	Previous Resection

1	26	4	Male	Parietal-temporal	No
2	44	12	Male	Occipitoparietal	No
3	52	26	Male	Frontotemporal	No
4	48	20	Male	Frontotemporal	Yes
5	51	10	Female	Occipitoparietal	No
6	50	15	Female	Frontotemporal	Yes
7	43	20	Male	Temporal	No
8	50	20	Male	Temporal	Yes
9	36	5	Male	Temporal	Yes

**TABLE II:** Next Seizure Prediction Performance Averaged Across Patients With a 24 Hours Cutoff for ISI. Cross-Validated Mean and Standard Deviation Are Given for Each Metric (in %)

Model	Precision	Recall	Fl-score	AUC

Log. Reg.	69.3 (23.5)	70.0 (16.0)	68.9 (14.4)	65.2 (7.7)
SVM	73.7 (18.6)	76.9(20.7)	73.4 (14.3)	55.4 (13.2)
KNN	**80.9 (13.9)**	75.6 (24.2)	**78.8 (14.8)**	61.7 (7.5)
Decision Tree	73.7 (23.3)	56.5 (15.7)	61.9 (15.8)	61.7 (8.3)
Random Forest	76.6 (19.0)	68.6 (14.9)	73.4 (14.6)	**69.5 (11.0)**
Baseline 1	62.5 (29.2)	50.0 (0.0)	52.3 (14.6)	50.0 (0.0)
Baseline 2	62.5 (29.2)	**100.0 (0.0)**	73.0 (25.1)	-(-)

**TABLE III:** Patient-Specific Next Seizure Prediction Performance for the Best Predictor With a 24 Hours Cutoff for ISI. Mean and Standard Deviation (in Parenthesis) Over Cross-Validation Is Provided for Each Metric (in %). Patient #2 Was Excluded From Prediction Analysis Because They Had Few Cluster-Non Last Seizures (*n* = 4)

Pt.	Sample Size		Random Forest				Baseline 1				Baseline 2			
	
#	I/C-L	C-NL	Precis.	Recall	FI	AUC	Precis.	Recall	FI	AUC	Precis.	Recall	FI	AUC

1	73	107	**66.9 (3.1)**	59.0 (12.6)	61.9 (7.8)	**61.2 (6.0)**	59.4	50.0	54.3	50.0	59.4	**100.0**	**74.6**	-
2	41	4	-	-	-	-	-	-	-	-	-	-	-	-
3	28	648	**97.2 (0.6)**	92.7 (1.9)	94.9 (0.9)	**78.5 (11.1)**	95.9	50.0	65.7	50.0	95.9	**100.0**	**97.9**	-
4	83	646	**90.2 (1.0)**	82.5 (5.4)	86.1 (3.0)	**54.8 (8.1)**	88.6	50.0	63.9	50.0	88.6	**100.0**	**94.0**	-
5	132	148	**57.9 (6.6)**	51.7 (10.9)	54.2 (8.5)	**57.7 (5.6)**	52.9	50.0	51.4	50.0	52.9	**100.0**	**69.2**	-
6	100	793	**91.2 (1.3)**	78.5 (5.2)	84.3 (3.0)	**64.8 (4.6)**	88.8	50.0	64.0	50.0	88.8	**100.0**	**94.1**	-
7	28	10	**85.0 (26.0)**	60.0 (37.4)	**72.6 (16.3)**	**89.0 (11.1)**	26.3	50.0	34.5	50.0	26.3	**100.0**	41.7	-
8	228	540	**81.7 (2.5)**	70.9 (2.1)	75.9 (1.6)	**72.1 (3.1)**	70.3	50.0	58.4	50.0	70.3	**100.0**	**82.6**	-
9	83	18	**42.4 (25.4)**	53.3 (26.7)	**57.6 (10.9)**	**78.3 (8.4)**	17.8	50.0	26.3	50.0	17.8	**100.0**	30.3	-

**TABLE IV:** Cluster Onset Prediction Performance for Each Model Averaged Across Patients With a 24 Hours Cutoff for ISI. Mean and Standard Deviation (in Parenthesis) Over Cross-Validation Are Provided for Each Metric (in %)

Model	Precision	Recall	Fl-score	AUC

Log. Reg.	69.2 (15.5)	70.3 (9.6)	68.5 (4.7)	47.8 (11.5)
SVM	**70.4 (16.2)**	78.4(19.6)	70.8 (6.9)	**55.3 (13.2)**
KNN	67.2 (18.2)	72.7 (24.7)	69.3 (21.7)	50.9 (7.5)
Decision Tree	68.9 (16.4)	60.5 (7.5)	60.2 (5.2)	52.0 (8.3)
Random Forest	67.7 (17.5)	70.1 (19.8)	68.5 (18.8)	51.3 (11.0)
Baseline 1	65.9 (15.6)	50.0 (0.0)	56.2 (5.7)	50.0 (0.0)
Baseline 2	65.9 (15.6)	**100.0 (0.0)**	**78.6 (11.1)**	-(-)

**TABLE V:** Patient-Specific Cluster Onset Prediction Performance for the Best Predictor With a 24 Hours Cutoff for ISI. Mean and Standard Deviation (in Parenthesis) Over Cross-Validation Is Provided for Each Metric (in %). Patients With Fewer Than 10 Isolated or Cluster-First Seizures Were Excluded From the Prediction Analysis

Pt.	Sample Size		SVM				Baseline 1				Baseline 2			
	
#	I	C-F	Precis.	Recall	FI	AUC	Precis.	Recall	FI	AUC	Precis.	Recall	FI	AUC

1	31	42	57.1 (0.0)	**100.0 (0.0)**	72.7 (0.0)	**54.2 (14.3)**	**57.5**	50.0	53.5	50.0	**57.5**	**100.0**	**73.0**	-
2	37	4	-	-	-	-	-	-	-	-	-	-	-	-
3	6	22	-	-	-	-	-	-	-	-	-	-	-	-
4	13	70	**88.6 (6.5)**	71.4 (7.8)	78.5 (4.1)	45.7 (24.5)	84.3	50.0	62.8	**50.0**	84.3	**100.0**	**91.5**	-
5	65	67	**51.7 (2.5)**	96.9 (3.8)	**67.4 (2.4)**	39.7 (10.4)	50.8	50.0	50.4	**50.0**	50.8	**100.0**	67.3	-
6	19	81	**84.2 (4.5)**	70.0 (20.7)	74.6 (11.9)	**67.1 (25.5)**	81.0	50.0	61.8	50.0	81.0	**100.0**	**89.5**	-
7	22	6	-	-	-	-	-	-	-	-	-	-	-	-
8	101	127	**70.5 (7.0)**	53.6 (11.8)	60.6 (10.1)	**70.1 (6.7)**	55.7	50.0	52.7	50.0	55.7	**100.0**	**71.5**	-
9	75	8	-	-	-	-	-	-	-	-	-	-	-	-

**TABLE VI:** Next Seizure Prediction Performance for Each Model Averaged Across Patients With an 8 Hours Cutoff for ISI. Mean and Standard Deviation (in Parenthesis) Over Cross-Validation Are Provided for Each Metric (in %)

Model	Precision	Recall	Fl-score	AUC

Log. Reg.	62.8 (20.5)	71.0 (4.5)	66.1 (13.1)	67.7 (6.1)
SVM	60.0 (19.9)	78.9 (23.2)	**69.8 (15.0)**	51.6 (14.8)
KNN	**72.8 (15.6)**	64.4 (25.8)	67.1 (21.1)	70.3 (10.4)
Decision Tree	64.2 (19.5)	59.4 (8.1)	59.5 (12.0)	65.2 (5.6)
Random Forest	67.3 (17.0)	59.3 (9.1)	64.7 (13.1)	**70.3 (5.8)**
Baseline 1	48.2 (25.3)	50.0 (0.0)	45.8 (15.4)	50.0 (0.0)
Baseline 2	48.2 (25.3)	**100.0 (0.0)**	61.4 (24.6)	-(-)

**table VII:** Cluster Onset Prediction Performance for Each Model Averaged Across Patients With an 8 Hours Cutoff for ISI. Mean and Standard Deviation (in Parenthesis) Over Cross-Validation Are Provided for Each Metric (in %)

Model	Precision	Recall	Fl-score	AUC

Log. Reg.	52.7 (11.0)	70.4 (16.0)	59.0 (11.0)	61.4 (5.4)
SVM	51.2 (10.1)	74.6(20.2)	60.9 (12.0)	54.5 (7.5)
KNN	56.0 (5.9)	49.8 (14.5)	51.9 (10.1)	61.2 (6.8)
Decision Tree	**59.5 (12.0)**	59.6 (18.8)	56.9 (13.3)	64.0 (10.1)
Random Forest	57.0 (9.9)	60.0 (7.5)	57.6 (8.1)	**64.2 (8.5)**
Baseline 1	44.8 (12.1)	50.0 (0.0)	46.6 (6.8)	50.0 (0.0)
Baseline 2	44.8 (12.1)	**100.0 (0.0)**	**61.1 (11.5)**	-(-)

## References

[1] Haut SR, “Seizure clusters: Characteristics and treatment,” Current Opinion Neurol, vol. 28, no. 2, pp. 143–150, 2015. 10.1097/WCO.0000000000000177 25695133

[2] Sillanpää M and Schmidt D, “Seizure clustering during drug treatment affects seizure outcome and mortality of childhood-onset epilepsy,” Brain, vol. 131, no. 4, pp. 938–944, Feb. 2008. 18310680 10.1093/brain/awn037

[3] Gidal B, Klein P, and Hirsch LJ, “Seizure clusters, rescue treatments, seizure action plans: Unmet needs and emerging formulations,” Epilepsy Behav, vol. 112, Nov. 2020, Art. no. 107391. 10.1016/j.yebeh.2020.107391 32898744

[4] Saboo KV , “Individualized seizure cluster prediction using machine learning and ambulatory intracranial EEG,” in Proc. IEEE Int. Conf. Bioinf. Biomed. (BIBM), Dec. 2022, pp. 1157–1163. 10.1109/TNB.2023.3275037 PMC10702269 37163411

[5] Ferastraoaru V , “Termination of seizure clusters is related to the duration of focal seizures,” Epilepsia, vol. 57, no. 6, pp. 889–895, Jun. 2016. 27030215 10.1111/epi.13375

[6] Gregg NM , “Circadian and multiday seizure periodicities, and seizure clusters in canine epilepsy,” Brain Commun, vol. 2, no. 1, Jan. 2020, Art. no. fcaa008. 10.1093/braincomms/fcaa008 PMC7052793 32161910

[7] Karoly PJ, Nurse ES, Freestone DR, Ung H, Cook MJ, and Boston R, “Bursts of seizures in long-term recordings of human focal epilepsy,” Epilepsia, vol. 58, no. 3, pp. 363–372, Mar. 2017. 28084639 10.1111/epi.13636 PMC5339053

[8] Chen H-H, Shiao H-T, and Cherkassky V, “Online prediction of lead seizures from iEEG data,” Brain Sci, vol. 11, no. 12, p. 1554, Nov. 2021. 34942859 10.3390/brainsci11121554 PMC8699082

[9] Burns SP , “Network dynamics of the brain and influence of the epileptic seizure onset zone,” Proc. Nat. Acad. Sci. USA, vol. 111, no. 49, pp. E5321–E5330, Dec. 2014. 25404339 10.1073/pnas.1401752111 PMC4267355

[10] Zaveri HP , “Controversies on the network theory of epilepsy: Debates held during the ICTALS 2019 conference,” Seizure, vol. 78, pp. 78–85, May 2020. 32272333 10.1016/j.seizure.2020.03.010 PMC7952007

[11] Yaffe RB , “Physiology of functional and effective networks in epilepsy,” Clin. Neurophysiol, vol. 126, no. 2, pp. 227–236, Feb. 2015. 25283711 10.1016/j.clinph.2014.09.009

[12] Gidal B and Detyniecki K, “Rescue therapies for seizure clusters: Pharmacology and target of treatments,” Epilepsia, vol. 63, no. S1, pp. S34–S44, Sep. 2022. 35999174 10.1111/epi.17341 PMC9543841

[13] Brinkmann BH , “Forecasting seizures using intracranial EEG measures and SVM in naturally occurring canine epilepsy,” PLoS ONE, vol. 10, no. 8, Aug. 2015, Art. no. e0133900. 10.1371/journal.pone.0133900 PMC4524640 26241907

[14] Cook MJ , “Prediction of seizure likelihood with a long-term, implanted seizure advisory system in patients with drug-resistant epilepsy: A first-in-man study,” Lancet Neurol, vol. 12, no. 6, pp. 563–571, Jun. 2013. 23642342 10.1016/S1474-4422(13)70075-9

[15] Cimbalnik J , “Multi-feature localization of epileptic foci from interictal, intracranial EEG,” Clin. Neurophysiol, vol. 130, no. 10, pp. 1945–1953, Oct. 2019. 31465970 10.1016/j.clinph.2019.07.024 PMC6759804

[16] Saboo KV , “Leveraging electrophysiologic correlates of word encoding to map seizure onset zone in focal epilepsy: Task-dependent changes in epileptiform activity, spectral features, and functional connectivity,” Epilepsia, vol. 62, no. 11, pp. 2627–2639, Nov. 2021. 34536230 10.1111/epi.17067 PMC8563435

[17] Sladky V , “Distributed brain co-processor for tracking spikes, seizures and behaviour during electrical brain stimulation,” Brain Commun, vol. 4, no. 3, May 2022, Art. no. fcac115. [Online]. Available: 10.1093/braincomms/fcac115 PMC9217965 35755635

[18] Bower MR , “Reactivation of seizure-related changes to interictal spike shape and synchrony during postseizure sleep in patients,” Epilepsia, vol. 58, no. 1, pp. 94–104, Jan. 2017. 27859029 10.1111/epi.13614 PMC5358812

[19] Chiang S , “Individualizing the definition of seizure clusters based on temporal clustering analysis,” Epilepsy Res, vol. 163, Jul. 2020, Art. no. 106330. 10.1016/j.eplepsyres.2020.106330 32305858

[20] Stirling RE, Cook MJ, Grayden DB, and Karoly PJ, “Seizure forecasting and cyclic control of seizures,” Epilepsia, vol. 62, no. S1, pp. 2–14, Feb. 2021. 10.1111/epi.16541 32712968

[21] Nejedly P , “Deep-learning for seizure forecasting in canines with epilepsy,” J. Neural Eng, vol. 16, no. 3, Jun. 2019, Art. no. 036031. 10.1088/1741-2552/ab172d 30959492

[22] Brinkmann BH , “Crowdsourcing reproducible seizure forecasting in human and canine epilepsy,” Brain, vol. 139, no. 6, pp. 1713–1722, Jun. 2016. 27034258 10.1093/brain/aww045 PMC5022671

[23] Maturana MI , “Critical slowing down as a biomarker for seizure susceptibility,” Nature Commun, vol. 11, no. 1, pp. 1–12, May 2020. 32358560 10.1038/s41467-020-15908-3 PMC7195436

[24] Truong ND , “Convolutional neural networks for seizure prediction using intracranial and scalp electroencephalogram,” Neural Netw, vol. 105, pp. 104–111, Sep. 2018. 29793128 10.1016/j.neunet.2018.04.018

[25] Jafarpour S, Hirsch LJ, Gaínza-Lein M, Kellinghaus C, and Detyniecki K, “Seizure cluster: Definition, prevalence, consequences, and management,” Seizure, vol. 68, pp. 9–15, May 2019. 29871784 10.1016/j.seizure.2018.05.013

[26] Seneviratne U, Karoly P, Freestone DR, Cook MJ, and Boston RC, “Methods for the detection of seizure bursts in epilepsy,” Frontiers Neurol, vol. 10, p. 156, Feb. 2019. 10.3389/fneur.2019.00156 PMC6400839 30873108

[27] Ilyas A , “Forecasting seizure clusters from chronic ambulatory electrocorticography,” Epilepsia, vol. 63, no. 9, pp. e106–e111, Sep. 2022. 35751497 10.1111/epi.17347

[28] Bialonski S and Lehnertz K, “Assortative mixing in functional brain networks during epileptic seizures,” Chaos, Interdiscipl. J. Nonlinear Sci, vol. 23, no. 3, Sep. 2013, Art. no. 033139. 10.1063/1.4821915 24089975

[29] Chen H-H and Cherkassky V, “Performance metrics for online seizure prediction,” Neural Netw, vol. 128, pp. 22–32, Aug. 2020. 32387921 10.1016/j.neunet.2020.04.022 PMC7340210

[30] Rungratsameetaweemana N, Lainscsek C, Cash SS, Garcia JO, Sejnowski TJ, and Bansal K, “Brain network dynamics codify heterogeneity in seizure evolution,” Brain Commun, vol. 4, no. 5, Sep. 2022, Art. no. fcac234. 10.1093/braincomms/fcac234 PMC9527667 36196085

[31] Schroeder GM , “Seizure pathways change on circadian and slower timescales in individual patients with focal epilepsy,” Proc. Nat. Acad. Sci. USA, vol. 117, no. 20, pp. 11048–11058, May 2020. 32366665 10.1073/pnas.1922084117 PMC7245106

[32] Cao Y , “A transfer learning-based model for individualized clustered seizure prediction using intracranial EEG,” in Proc. 11th Int. IEEE/EMBS Conf. Neural Eng. (NER), Baltimore, MD, USA, 2023, pp. 1–4, doi: 10.1109/NER52421.2023.10123862.

